# Therapeutic Value of Drugs Frequently Marketed Using Direct-to-Consumer Television Advertising, 2015 to 2021

**DOI:** 10.1001/jamanetworkopen.2022.50991

**Published:** 2023-01-13

**Authors:** Neeraj G. Patel, Thomas J. Hwang, Steven Woloshin, Aaron S. Kesselheim

**Affiliations:** 1 Program on Regulation, Therapeutics, and Law (PORTAL), Division of Pharmacoepidemiology and Pharmacoeconomics, Department of Medicine, Brigham and Women’s Hospital and Harvard Medical School, Boston, Massachusetts; 2 Yale School of Medicine, New Haven, Connecticut; 3 Center for Medicine in the Media, Dartmouth Institute for Health Policy and Clinical Practice, Geisel School of Medicine at Dartmouth, Lebanon, New Hampshire; 4 Lisa Schwartz Foundation for Truth in Medicine, Norwich, Vermont

## Abstract

This cohort study assesses whether drugs with the most direct-to-consumer television advertising represent advances over existing treatments.

## Introduction

Direct-to-consumer drug advertising increased by nearly 5-fold from 1997 to 2016, with 663 000 television commercials reported in 2016. Television advertisements account for roughly two-thirds of total direct-to-consumer advertising spending.^1^ Direct-to-consumer advertising is associated with use of higher-cost drugs over generics and less expensive alternatives.^2^ Proponents have argued that such advertising improves public health by promoting clinically beneficial prescribing. We assessed the therapeutic value (ie, whether they represent advances over existing treatments) of drugs subject to most direct-to-consumer television advertising from 2015 to 2021.

## Methods

We collected monthly lists of the US top-advertised drugs and advertisements by manufacturer spending in direct-to-consumer television advertising from an industry publication (September 2015 to August 2021) and extracted advertised indications from an online advertising database.^3,4^ After excluding duplicates and advertisements without any mentioned products, we merged this list of advertised drug indications with television advertising spending data from Kantar Media AdSpender database and adjusted for inflation (2021 US dollars). The eMethods and eFigure in Supplement 1 describe this process and illustrate the inclusions and exclusions that arrived at our final cohort of 81 drugs.

For each drug indication, we obtained therapeutic value ratings from independent health technology assessment agencies of Canada, France, and Germany and defined ratings of moderate or greater therapeutic value as high therapeutic value, using methods described previously.^5^ Ratings consider drugs’ added benefit, safety, and strength of evidence, as compared with existing therapies. We used the most favorable rating available in cases of reevaluations or multiple ratings for a single indication (eg, for different patient subgroups). The final cohort included drugs with at least 1 therapeutic value rating.

We first assessed the proportion of commonly advertised drugs rated as high therapeutic value by any agency. We then examined drugs that were rated by at least 2 agencies. Analyses were conducted using Excel version 16.67 (Microsoft Corp). The study was exempt from institutional review board review and the requirement for informed consent, as no patient data were used. We followed the STROBE reporting guideline.

## Results

Among the 81 top-advertised drugs, 26 (32.1%) were immunomodulating agents; 13 (16.0%), alimentary tract and metabolism drugs; and 11 (13.6%) neurologic drugs. Among these, 73 (90.1%) had at least 1 therapeutic value rating, and 55 (67.9%) had ratings from at least 2 health agencies. The 73 drugs with at least 1 therapeutic value rating were associated with advertising spending of $22.3 billion from 2015 to 2021 (Table). Twenty of these commonly marketed drugs (27.4%), representing $6.4 billion (28.7%) in advertising spending, were rated by any agency as having high therapeutic value. Results were similar in the subgroup of drugs with at least 2 ratings: 17 of 55 drugs (30.9%) were rated as having high therapeutic value, accounting for 32.7% of advertising spending.

**Table.** Television Advertising Spending and Therapeutic Value for Top Advertised Drugs With at Least 1 Therapeutic Value Rating^a^

Characteristic	All included drugs (n = 73)	Drugs categorized as high benefit (n = 20)	Drugs categorized as low benefit (n = 53)
TV advertising spending, 2015-2021, $ in billions (% total)	22.3 (100.0)	6.4 (28.7)	15.9 (71.3)
Median (IQR) TV ad spending 2015-2021, $ in millions	265.8 (163.5-457.2)	338.4 (250.2-520.4)	231.7 (136.4-410.5)
Top advertised drugs by dollar amount spent (indication)	Dulaglutide (type 2 diabetes) Varenicline (smoking cessation) Tofacitinib (rheumatoid arthritis)	Adalimumab (psoriatic arthritis) Sacubitril/valsartan (heart failure) Pembrolizumab (lung cancer)	Dulaglutide (type 2 diabetes) Varenicline (smoking cessation) Tofacitinib (rheumatoid arthritis)

^a^ Dollar values are adjusted for inflation (2021 US dollars). Total television advertising spending data were obtained from Kantar Media AdSpender database. Beginning in 2018, the Kantar advertising data incorporated a new source of national cable television advertising data.

## Discussion

Fewer than one-third of the most common drugs featured in direct-to-consumer television advertising were rated as having high therapeutic value, defined as providing at least moderate improvement in clinical outcomes compared with existing therapies. Manufacturers’ television advertising spending on included products rated as low therapeutic value was $15.9 billion from 2015 to 2021. A limitation of this study was that a small number of drugs lacked ratings, although these only accounted for 5% of advertising spending. We used the most favorable therapeutic value rating at any point since approval, which may overestimate the proportion of higher-benefit drugs.

The findings of this study are consistent with prior research that questioned the therapeutic value of drugs heavily promoted to clinicians.^6^ One explanation might be that drugs with substantial therapeutic value are likely to be recognized and prescribed without advertising, so manufacturers have greater incentive to promote drugs of lesser value. The American Medical Association and public health advocates have called for restrictions on direct-to-consumer drug advertising, warning that it inflates demand for newer, more expensive drugs at the expense of less costly alternatives. Policy makers and regulators could consider limiting direct-to-consumer advertising to drugs with high therapeutic or public health value or requiring standardized disclosure of comparative effectiveness and safety data, but policy changes would likely require industry cooperation or face constitutional challenge.

## Notes

Supplement 1.
eMethods.
**eFigure.** Identification of Advertised Drug-Indications
eReferences.

Supplement 2.
Data Sharing Statement
