# Supplementary material for: Therapeutic Value of Drugs Frequently Marketed Using Direct-to-Consumer Television Advertising, 2015 to 2021
Source: JAMA Netw Open. 2023 Jan 13;6(1):e2250991. doi: 10.1001/jamanetworkopen.2022.50991 (PMC9857401; doi:10.1001/jamanetworkopen.2022.50991)
Supplement: Supplement 1. — eMethods. eFigure. Identification of Advertised Drug-Indications eReferences. [file jamanetwopen-e2250991-s001.pdf]

Supplemental Online Content

Patel NG, Hwang TJ, Woloshin S, Kesselheim AS. Therapeutic value of drugs frequently marketed using direct-to-consumer television advertising, 2015 to 2021. *JAMA Netw Open.* 2023;6(1):e2250991. doi:10.1001/jamanetworkopen.2022.50991

eMethods.

eFigure. Identification of Advertised Drug-Indications

eReferences.

This supplemental material has been provided by the authors to give readers additional information about their work.

© 2023 Patel NG et al. *JAMA Network Open.*

eMethods.

Identification of top-advertised drugs and advertisements was performed by first reviewing monthly lists published by FiercePharma.[1] These lists include the ten pharmaceutical television advertising campaigns with the highest level of television advertising spending for the respective month, as well as the name of the advertisement with the highest associated spending total for each campaign. After excluding unbranded advertisements, we extracted each drug name that appeared in these lists from September 2015 – August 2021.

To extract indications, we pasted the name of each entry's advertisement into the search tool of the iSpot.tv advertising database. One investigator (N.G.P.) watched each advertisement and extracted the advertised indication. We then generated a list of unique drug-indications. **Supplementary Figure 1** describes our screening protocol.

One investigator (T.J.H) extracted therapeutic value ratings from the health technology assessment agencies of Canada, France, and Germany. As compared to a prior analysis by our group, the current analysis excluded Italy because of changes in availability of Italian data during the study period.[2] We used each drug-indication's most favorable rating in cases of re-evaluations or when a heath technology assessment agencies provided multiple ratings for a single drug-indication like for different patient subgroups.

We then used the Kantar Ad$pender database to obtain annual TV advertising spending for each product, stratified by indication, from September 2015 – August 2021. The spending figures were then linked to our initial list by drug-indication using Microsoft Excel. Data collection, including extraction of drug names, indications, spending data, and therapeutic value ratings, began in March 2021 and was completed in January 2022.

© 2023 Patel NG et al. *JAMA Network Open.*

eFigure. Identification of Advertised Drug-Indications

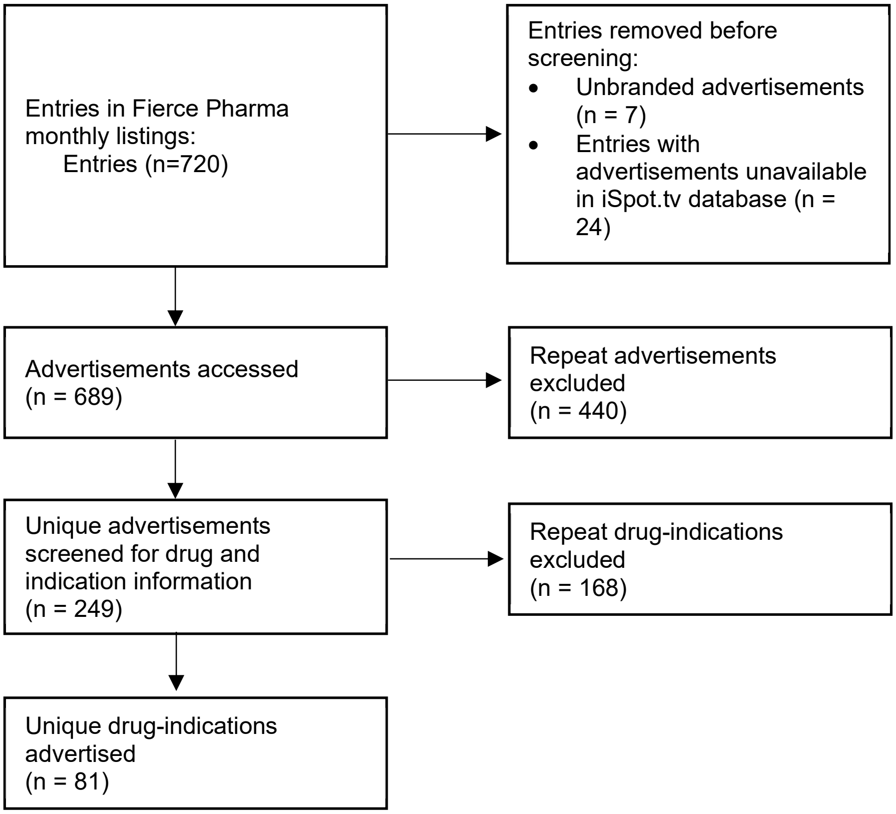

© 2023 Patel NG et al. *JAMA Network Open.*

eReferences.

[1] Bulick, B.S. Pharma TV spending jumps in November, with top brands scaling $200M. *Fierce Pharma*. Dec 10, 2020. Accessed October 20222. https://www.fiercepharma.com/marketing/pharma-tv-spending-jumps-november-top-brands-scaling-200-million

[2] Hwang TJ, Ross JS, Vokinger KN, Kesselheim AS. Association between FDA and EMA expedited approval programs and therapeutic value of new medicines: retrospective cohort study. BMJ 2020;371:m3434.

© 2023 Patel NG et al. *JAMA Network Open.*
