# Supplementary material for: Therapeutic Value of Drugs Frequently Marketed Using Direct-to-Consumer Television Advertising, 2015 to 2021
Source: JAMA Netw Open. 2023 Jan 13;6(1):e2250991. doi: 10.1001/jamanetworkopen.2022.50991 (PMC9857401; doi:10.1001/jamanetworkopen.2022.50991)
Supplement: Supplement 2. — Data Sharing Statement [file jamanetwopen-e2250991-s002.pdf]

Data Sharing Statement

Patel. Therapeutic Value of Drugs Frequently Marketed Using Direct-to-Consumer Television Advertising, 2015 to 2021. *JAMA Netw Open*. Published January 13, 2023. doi:10.1001/jamanetworkopen.2022.50991

Data
Data available: No
